# The critical elements of effective academic-practice partnerships: a framework derived from the Department of Veterans Affairs Nursing Academy

**DOI:** 10.1186/s12912-014-0036-8

**Published:** 2014-12-20

**Authors:** Aram Dobalian, Candice C Bowman, Tamar Wyte-Lake, Marjorie L Pearson, Mary B Dougherty, Jack Needleman

**Affiliations:** Veterans Emergency Management Evaluation Center (VEMEC), North Hills, CA USA; HSR&D Center for the Study of Healthcare Innovation, Implementation and Policy, (CSHIIP), 16111 Plummer St. MS-152, Sepulveda, North Hills, CA USA; Department of Health Policy and Management, University of California, Los Angeles School of Public Health, 650 Charles Young Dr. S., Room 31-236B CHS, Los Angeles, CA 90095-1772 USA; University of California, Los Angeles School of Nursing, Los Angeles, CA USA; RAND Corporation, 1776 Main Street, Santa Monica, CA 90401-3208 USA; Department of Veterans Affairs, Office of Academic Affiliations, 1800 G Street NW, Washington, DC USA

## Abstract

**Background:**

The nursing profession is exploring how academic-practice partnerships should be structured to maximize the potential benefits for each partner. As part of an evaluation of the U.S. Department of Veterans Affairs Nursing Academy (VANA) program, we sought to identify indicators of successful partnerships during the crucial first year.

**Methods:**

We conducted a qualitative analysis of 142 individual interviews and 23 focus groups with stakeholders from 15 partnerships across the nation. Interview respondents typically included the nursing school Dean, the VA chief nurse, both VANA Program Directors (VA-based and nursing school-based), and select VANA faculty members. The focus groups included a total of 222 VANA students and the nursing unit managers and staff from units where VANA students were placed. An ethnographic approach was utilized to identify emergent themes from these data that underscored indicators of and influences on Launch Year achievement.

**Results:**

We emphasize five key themes: the criticality of inter-organizational collaboration; challenges arising from blending different cultures; challenges associated with recruiting nurses to take on faculty roles; the importance of structuring the partnership to promote evidence-based practice and simulation-based learning in the clinical setting; and recognizing that stable relationships must be based on long-term commitments rather than short-term changes in the demand for nursing care.

**Conclusions:**

Developing an academic-clinical partnership requires identifying how organizations with different leadership and management structures, different responsibilities, goals and priorities, different cultures, and different financial models and accountability systems can bridge these differences to develop joint programs integrating activities across the organizations. The experience of the VANA sites in implementing academic-clinical partnerships provides a broad set of experiences from which to learn about how such partnerships can be effectively implemented, the barriers and challenges that will be encountered, and strategies and factors to overcome challenges and build an effective, sustainable partnership. This framework provides actionable guidelines for structuring and implementing effective academic-practice partnerships that support undergraduate nursing education.

## Background

In a business context, strategic alliances may be conceptualized as formal or informal collaborative relationships between parties who come together to achieve a common goal while remaining separate organizations [1]. In the nursing profession, strategic alliances are typically created between an academic partner or nursing school and a practice partner, usually an inpatient healthcare facility, that are geographically proximate to each other. The potential benefits of such alliances, or “academic-practice partnerships,” may include increased capacity for students to matriculate into nursing programs [2] and the ability to better prepare nursing students to meet the demands of the practice setting [3]. Medicine has benefited from such academic-practice partnerships for decades, but nursing is comparatively new to the academic-practice partnership model [4].

The Department of Veterans Affairs (VA), as a major employer of nurses and other healthcare professionals, has an abiding interest in clinician education, and, while not an educational institution itself, does make its facilities available for clinical instruction. Moreover, for disciplines such as medicine, VA has been a major employer of clinical teaching staff and an active participant in shaping clinical experiences. VA recently extended this academic-practice partnership framework to nursing education on a pilot basis. In 2007, the VA Offices of Academic Affiliations and Nursing Services established the VA Nursing Academy (VANA), a five-year, $60 million pilot program, to address the nursing and nurse faculty shortage occurring both within VA and the nation by providing pilot funding for 15 strategic partnerships to be established between competitively selected VA facilities and nearby nursing schools nationwide [5].

The VANA partnership model was designed to develop strong, mutually beneficial relationships between participating nursing schools and VA facilities by (1) expanding educational faculty through career development, (2) increasing student enrollments, (3) cultivating educational and practice innovations, and (4) increasing recruitment and retention of VA nurses as a result of enhanced roles in nursing education. In addition, the VANA model was designed to allow for substantial local variation in its structure and application. This approach afforded each partnership with the ability to adapt the program to meet local contextual needs and demands, and also permitted significant experimentation to ascertain the relative success of various approaches.

The nursing profession is currently exploring how such arrangements may be most effectively operationalized to maximize the potential benefits for each partner and for the field. The nursing literature provides many examples of partnerships that include one nursing school and one or more practice partners, but the evidence for the success of these partnerships is poor [6]. Many of these articles describe the development and nature of the partnership model, but collectively they provide little empirical evidence to guide organizational decision-makers when they seek to design new partnerships or modify existing ones [7–14]. Recent recommendations from a task force established by the American Association of Colleges of Nursing (AACN) and the American Organization of Nurse Executives (AONE) on academic-practice partnerships [15] provide some guideposts for such partnerships, but they are guiding principles rather than recommended practices for their implementation.

With five years of funding for fifteen partnerships, VANA provided an oppportunity to increase the evidence base on the implementation effectiveness of partnerships. Towards this end, the VA funded a six-year, multi-dimensional evaluation of the VANA program. As part of this evaluation, the evaluation team sought to identify specific indicators of successes and challenges that characterized VANA partnerships during the “start-up phase” of each partnership. This article proposes a framework of critical features that have emerged from our qualitative data analysis during an examination of the first year, or “Launch Year,” of each partnership. In so doing, we propose certain features as early, evidence-based indicators of progress toward achieving desired outcomes, and provide practical, real-world details that extend the guiding principles in the aforementioned AACN-AONE report. These findings, drawn from a comparative analysis of partnership development at 15 sites from across the United States, provide critical insight into effective approaches for structuring and implementing academic-clinical partnerships in nursing.

This article addresses the following two research questions:

1. What implementation activities and goal-specific outputs were associated with a successful launch?
2. What program inputs and contextual factors also were associated with a successful launch?

## Methods

**Figure 1:**
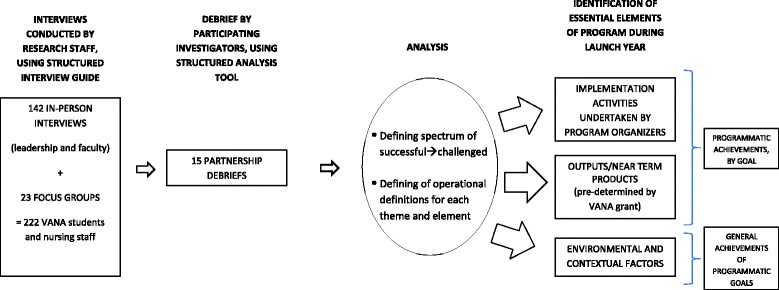
**Methods flow chart.**

This formative evaluation [16] was designed to qualitatively analyze data collected using in-person interviews and focus groups from 364 key informants involved in implementing these 15 partnerships. As Figure 1 illustrates, an ethnographic approach was utilized to identify emergent themes from these data that underscored indicators of and influences on Launch Year achievement.

Data collection included 142 in-person interviews with faculty and administrators during prescheduled site visits to each locale and 23 focus groups with nursing staff and students. Each site was visited one time within the first 18 months of joining the VANA pilot. Interview respondents typically included the nursing school Dean, the VA chief nurse, both VANA Program Directors (VA-based and nursing school-based), and select VANA faculty members. The focus groups included a total of 222 VANA students and the nursing unit managers and staff from units where VANA students were placed. Both the interviews and the focus groups included open-ended questions concerning the general background of the institutions and their motivation to participate in VANA, various structural and operational aspects of the partnership, opinions about the potential effectiveness and possible impacts of VANA on the respondent and their organization, and perceptions regarding the effectiveness of VANA in improving the VA’s ability to recruit new graduates. Each interview lasted approximately 60 to 90 minutes; the focus groups were typically 60 minutes. Two to four doctorally-prepared investigators were present at each interview or focus group session with one investigator serving as the primary interviewer, and the other(s) keeping detailed field notes, verifying observations, and ensuring that all key topics were covered [17]. All interviews and focus groups were audiotaped. Participants were informed that data was kept confidential and anonymous. Verbal informed consent for participation was obtained from participants. This study was approved by the Institutional Review Board of the VA Greater Los Angeles Healthcare System.

The research team used a structured tool, derived from the interview guide and the team’s collective expertise and ongoing experience, to organize and analyze the interview and focus group data. Directly after each site visit, the investigators who participated in the interviews met and debriefed each other using this tool to capture impressions and comments in a timely manner. Investigators often referred to their field notes during this process. Recordings, notes and transcriptions were used to settle disagreements until consensus was reached for each question in the analysis tool.

Our qualitative analyses focused on identifying the essential elements of program progress during the Launch Year. We defined progress as achieving the programmatic goals designated for VANA by VA. For each goal, we identified two categories of elements that influenced success: implementation activities undertaken by the program organizers and outputs or near term products (often predetermined by the VANA grant).

We searched for variability within each element that would indicate a spectrum of progress ranging from “proceeding well according to plan” (i.e. successful) to “addressing impediments and therefore lagging behind schedule” (i.e. challenged). Operational definitions for each theme and element, and observed indicators of success were collectively drawn from the data. Disagreements among the analysts were settled by consensus. Note that the indicators under each of the domains are not structured to strictly parallel each other; the data are presented in this manner to limit the size of the tables. Thus, the presence or absence of a particular indicator generally characterizes whether a partnership may be considered successful or challenged within that domain.

When analyzing these implementation activities and outputs critical to successful launch, it should be noted that the initial inputs into the program and the program’s environmental context also influenced the partnership’s launch. We applied a similar qualitative approach to analyzing these elements, with progress being defined as achieving programmatic goals more generally, rather than specifically for each goal.

## Results

Tables 1, 2, 3, 4 and 5 present the critical implementation activities and outputs that were associated with a successful launch of the partnership, as well as with a challenged launch, by each of the five VANA goals.

**Table 1:** **Critical elements: implementation activities and outputs for increasing faculty positions**

**Critical element**	**Indicators of success**	**Indicators of challenge**
***Activities***		
*Define scope of faculty roles*	● Responsibilities of faculty positions were commensurate with FTE allotted to each position	● Responsibilities of new positions were not feasible within the allotted FTEs
	● Candidates were selected based on clinical expertise and previous teaching experience	● New faculty had little or no prior clinical or didactic teaching experience
	● New hires were willingly assigned to teach in some didactic courses, in addition to clinical instruction, in some cases, despite lack of previous experience	● No clear reporting structure delineated for VANA faculty
	● VANA faculty were generally well known to VA nursing staff before VANA launch	
	● Protected time from teaching was offered to new hires to engage in other activities (e.g., faculty meetings, committee work)	
*Initiate training for faculty role/faculty development*	● Ongoing mentoring was offered to new faculty, who appreciated its benefits	● No mentoring was offered to new faculty, particularly for those with little to no previous teaching experience
	● Learning opportunities with pedagogical focus provided that met different levels of skills and experiences of new hires	
		● Brief or no faculty orientation was offered
	● [Indicator of *partial* success] One-time faculty orientation session provided that did or did not include content on teaching methods	● New faculty were required to use teaching software (e.g., Blackboard) without sufficient training or support
		● VA-based faculty were not provided teaching materials (e.g., textbooks) in a timely manner
*Plan to integrate faculty into partnering environment*	● Each partnering organization welcomes involvement of VANA faculty in participating in department concerns	● VANA faculty are not considered as resources in addressing problems or developing new programs in nursing departments
*Adjust faculty workloads as needed*	● New faculty coped with teaching assignments with ease and enthusiasm	● Clinical groups had over 10 students
	● Clinical group size (i.e., typically 8–10 students or less) allowed for adequate student interactions with faculty	● New faculty had no access to information on teaching tips (e.g., grading care plans efficiently) that would have prevented them from feeling overwhelmed with workload
	● Faculty were paid for all hours worked or received “comp time” for grading at home	
	● Support was provided for faculty who needed more assistance in coping with workload during Launch Year	● VA-based faculty were re-assigned to old clinical, administrative, or educational responsibilities in VA when classes were not in session
	● During breaks, VA-based faculty worked on ancillary programs such as curriculum development, both at the VA and nursing school	● VA-based faculty, who held full-time VA positions, had no vacation breaks at the end of terms as did the school-based faculty
*Integrate faculty into partnering environment*	● VANA faculty group was very cohesive and supportive of each other, regardless of where they were based (i.e., VA or school)	● Among partnership members, there existed a lack of awareness of each other’s responsibilities and contributions
	● VA-based faculty become involved in nursing school committees where their clinical expertise is welcomed (e.g., in curriculum development)	● Clinical Expertise of VA-based faculty is not recognized or utilized by the members of the partnering institution
	● Nursing school-based faculty become involved in VA committees, particularly EBP	● Faculty felt that they had two masters (i.e., were beholden to the demands of both the nursing school and the VAMC)
	● VA-based faculty were engaged in university activities depending on interests and role requirements	
	● VA-based faculty perceived that their contributions were highly valued by nursing school colleagues	● Faculty felt like ‘outsiders’ when in partnering institution
	● VANA faculty had a high sense of collegiality with VA and nursing school colleagues	
*Give APRN faculty protected time for patient care*	● Partnership leaders were aware of the value of having VA APRNs as clinical faculty	● APRN faculty worked as care providers outside of full time position, often outside the VA, to maintain licensure or certification
	● Release time provided for APRN faculty to provide direct care to meet licensure or certification requirements	
***Outputs***		
*Faculty hiring quota met by end of Launch Year*	● Hiring quotas for VANA faculty positions met by at least the end of the first academic year, if not before	● Full hiring quota was not met by end of the first year, often due to scarcity of qualified (e.g., masters-prepared) applicants in local area
	● [*Partial* success] Faculty hiring quotas were met but new hires retained some or all of old responsibilities related to their previous positions	
	● Faculty assignments were made according to experience, expertise, interests, and programmatic needs	
*Faculty satisfied with new roles*	● Minimal turnover of faculty	● Some faculty left (or were asked to leave) positions by end of first year
	● If there was turnover in faculty, it was usually associated with personal circumstances	
	● High levels of faculty satisfaction were measured in VNEP surveys (e.g., with mentorship, leadership support, availability of teaching resources)	● Turnover often due to discontent resulting from unrealistic expectations about the role and its associated workload
		● High levels of faculty dissatisfaction measured in VNEP surveys

**Table 2:** **Critical elements: implementation activities and outputs for increasing student enrollment**

**Critical element**	**Indicators of success**	**Indicators of challenge**
***Activities***		
*Generate interest in VA clinical placements amongst students*	● In sites where students select clinical placements, VA representatives provided engaging presentations regarding the benefits of interning at the VA	● Disgruntled students placed at VA for clinical rotations; Concerns raised about being placed in an older, predominately male environment
*Place students on VAMC units*	● Processing of students through human resources (HR) at VAMC went smoothly and usually occurred prior to first day of clinical placements	● Student placements on VA units were delayed, often until late in the first semester, due to lack of anticipation of cumbersome VA HR policy requirements
	● [Indicator of *partial* success] Students often spent their initial clinical placement day or days completing HR processes and orienting to the VAMC	
***Outputs***		
*Increase undergraduate student enrollment*	● All or nearly all of increased student enrollment quotas (per grant requirements) were met by the beginning of the first school year	● Enrollment quotas were not met by end of first year
		● Significant logistical hurdles were encountered in orienting students into newly offered curriculum
	● [Indicator of *partial* success] Increased student quotas were met, but enrollments were made into a newly offered curriculum (e.g., 12-month accelerated program) that often still had unresolved logistical hurdles	
*Increased student satisfaction with participation*	● High levels of student satisfaction were measured in VNEP surveys	● High levels of student dissatisfaction were measured in VNEP surveys
	● Students often competed for slots in programs where the option to have all clinical placements at the VAMC (except pediatrics and obstetrics) was offered	● Students were required to have all clinical placements (except pediatrics and obstetrics) at the VAMC without a choice of healthcare facilities

**Table 3:** **Critical elements: implementation activities and outputs for implementing curricular innovations**

**Critical element**	**Indicators of success**	**Indicators of challenge**
***Activities***		
*Operationalize proposed innovations*	● Sufficient office space was provided for faculty to prepare for clinical sessions, grade assignments and meet with students	● There was a lack of dedicated office space for VANA faculty at either institution
	● Computer access and email accounts were provided to all partnership personnel at both the VAMC and the nursing school	● Ad hoc availability of non-private space at the VAMC was often the only option for faculty to meet with students
	● Meeting space was provided for partnership personnel to conduct regularly held partnership meetings	● Only limited or inconvenient access to email accounts was provided
	● Necessary instructional resources (e.g., textbooks and other teaching materials) were provided to faculty	● Only limited parking was available at one or both locales, which made commuting between institutions difficult
*Initiate proposed innovations*	● Program launch at the beginning of the first academic year was well-planned and staged from time of grant notification	● There were significant deviations from the proposed launch schedule by the end of year one
	● Program launch process mostly kept to schedule delineated in partnership’s proposal	● Limited evidence of proposed innovations being implemented by end of first year, often due to the continuing distraction of coping with unforeseen logistical barriers since launch (e.g., faculty and/or leadership turnover)
	● Nontraditional care areas of the VAMC (e.g., ambulatory mental health clinics) were used for some clinical placements	
	● Clinical experiences often included home health or outpatient clinics that focused on care continuity and the whole patient (e.g., co-morbid conditions, social situations)	● Neither partner seemed to recognize unique clinical teaching opportunities available within the VAMC (e.g., use of mental health units)
	● Presence of VANA program facilitated creation of or bolstered existing VA student nurse apprenticeship programs (e.g., pre-baccalaureate residency, other VA programs)	
	● DEU-style learning units were developed specifically for VANA clinical placements	
	● Scope of student experiences was increased on some units, particularly where clinical faculty was well-known to nursing staff as a colleague	
	● Simulation Lab resources and use, often at both facilities, were expanded to enhance VANA student learning	
	● Curricular content, both didactic and simulation, was infused with veteran-specific content and case studies	
*Collaborate on research and quality improvement initiatives*	● At least one of the program directors has strong research background and expertise	● No clear plans exist for collaborative research or QI projects between partners
	● QI projects are based on needs identified at the unit level	● No attempt to engage nursing staff in QI initiatives
	● Embedding VANA faculty on particular units facilitates implementation of QI projects	
	● VANA faculty are members of VA evidence-based practice committees	
*Refine program components as needed*	● Partnership conducted local site evaluation	● No local site evaluation activities conducted
	● Partnership had planned measurement strategy to use as feedback in modifying program	● Little evidence of any performance monitoring in place to refine program
***Outputs***		
*Increased stakeholder satisfaction with participation*	● Nursing staff on units used for VANA clinical placements eager to teach students	● Presence of VANA nursing students on units not viewed as a beneficial influence on delivery of care quality (perhaps even viewed as detrimental in some circumstances)
	● Veteran patients enthusiastic about having VANA nursing students provide their care	
*Increased evidence-based care*	● Unit nurses are actively involved in EBP journal clubs	● Weak or no attempt to integrate EBP changes into unit routines
	● EBP changes introduced by VANA faculty become institutionalized on certain units	
*Perceived improvements in nursing care quality*	● Improvements to patient care resulting from VANA innovations (e.g., DEU) recognized by nursing staff	● No influence of VANA innovations on patient care or on how VA units interact with nursing students

**Table 4:** **Critical elements: implementation activities and outputs for increasing recruitment and retention**

**Critical element**	**Indicators of success**	**Indicators of challenge**
***Activities***		
*Identify VA units to be used for VANA placements*	● Units struggling to accommodate nursing students receive increased mentorship and support by VANA faculty	● No VA units slated for VANA clinical placements viewed as being able to provide unique clinical learning opportunities
*Involve nursing staff in planning*	● VA-based faculty (i.e., staff nurses hired into faculty positions) highly integrated into workflow on units where students were placed	● Unit nursing staff as a whole not enthusiastic about having VANA students placed there
	● VA-based faculty highly engaged in all aspects of clinical teaching (e.g., preceptor training, DEU implementation)	● Presence of VANA students seen by unit staff as an increased workload burden and interruption to workflow
	● VANA faculty activated to develop their careers (e.g., furthering own education, gaining broad teaching experience)	● Unit staff not willing to participate in VANA-related activities, such as EBP projects
		● No formal clinical preceptor program was in place at VA facility
	● Benefits offered at some VAs encouraged RN nursing staff to further their education (e.g., tuition reimbursement, release time incentives, giving credit for precepting nursing students)	● VANA faculty minimally involved with clinical teaching, especially with large clinical groups
		● Over-reliance on nursing staff for clinical teaching, often beyond their training; often disrupting responsibilities of staff nurse
		● Too much time spent acclimating students to the unit environment at the expense of patient interactions
***Outputs***		
*Increased stakeholder satisfaction with participation*	● Clinical innovations resulting from VANA (e.g., DEU, embedded faculty) perceived to directly result in improved care quality	● Presence of VANA students is perceived as an increased burden and disruption
	● Unit nursing staff value input from VANA faculty	
	● Unit workflow viewed as improved with presence of VANA faculty and students	
*Benefits of VANA participation realized*	● VANA program provides clinically expert VA nurses teaching opportunities	● Unit nursing staff remain uninterested and unengaged in the placement of nursing students on their units
	● Nursing positions at VA provided for newly graduated VANA students	
	● Positive perceptions of VA clinical training improve VA’s reputation among subsequent groups of nursing students	● No positions exist at VA for newly graduating VANA students
		● Over-reliance on unit nursing staff for clinical teaching

**Table 5:** **Critical elements: implementation activities and outputs for promoting collaboration**

**Critical element**	**Indicators of success**	**Indicators of challenge**
***Activities***		
*Initiate communication structure*	● Pre-existing professional and/or personal relationships between leaders (i.e., Dean, Nurse Executive, Program Directors)	● Key leaders had never met
		● No recent history of interaction between partnering institutions
	● Prior and ongoing interaction between partnering institutions	● Significant disparity between benefits of the program to the partners
	● Parallel institutional missions (e.g., caring/educating the underserved), shared participation of objectives, and overt expectation of benefits overlap and complement	● No cross-institutional relationship existed between nursing leaders (e.g., Dean and Nurse Executive) and no recognition that such a relationship was necessary or beneficial
		● VA (or specifically VA Nursing Service) not respected by academic partner
*Create partnership governance (e.g., power sharing, problem solving)*	● Shared decision-making between partners	● Unilateral decision making by one side of the partnership or the other (e.g., determining selection criteria for faculty hires)
	● HR departments of both institutions works closely with partnership in processing new faculty and in preparing student nurses for clinical placements in VA	
	● IT departments in both institutions willing and able to resolve issues efficiently	● Antagonistic relationship in VA between service departments (e.g., nursing and staff education) over emerging issues related to VANA implementation, such as who oversees VANA program
*Elicit support for program from all levels of organizational leadership*	● Formal and regular standing meetings planned (and held) between:	● Planned formal meetings poorly attended, especially by core leaders
	→Program Directors	● Only interaction with OAA is through the scheduled program director calls despite presence of significant barriers to implementation
	→Both program directors and faculty	
	→Dean and Nurse Executive	
	→Dean, Nurse Executive, and both program directors	
	● Frequent ad hoc contacts (e.g., in-person, email, phone) between:	● Tensions between program directors and nurse leader(s) that either inhibit collaborative problem solving or introduce barriers
	→Program Directors	
	→Faculty members	
	→Dean, Nurse Executive, and program directors	
	● Dean and Nurse Executive regard themselves as colleagues	● Reluctance to contact OAA for advice and assistance in overcoming challenges that arise
	● Contacts with OAA, as necessary, outside of regularly scheduled program director conference calls	
*Delineate level of each program director’s involvement*	● Frequent, sometimes daily, informal contact between program directors to discuss and address program operations and issues	● At least one program director has minimal knowledge of program details and logistics
	● Program directors have awareness of details beyond broad objectives of program	● One program director less involved in day-to-day operations than counterpart
	● Both program directors have direct involvement in problem resolution	● A program director has limited respect and authority within own institution
	● Each program director has strong sense of ownership for program and feels directly responsible for its success	● Scope of VANA role exceeds time allotment
	● Program directors are actively involved in day-to-day activities	● A program director provides verbal support for program but has limited or no direct involvement
	● Program directors are held in high esteem by partnership and organizational colleagues	
	Each program director holds a position with high level of responsibility within institution	
	● Each program director often has long employment history with one or both partnering institutions	
	● Each program director has sufficient protected time to fulfill VANA role	
*Delineate level of Dean’s and Nurse Executive’s involvement*	● Both act as overseers and high level problem solvers for partnership	● Has minimal knowledge of program beyond its broadest objectives (e.g., being new to the position)
	● Both facilitate provision of institutional resources by lending authority of role	In cases where position turns over, newly hired leader views value of VANA differently than predecessor
	● Neither are involved in day-to-day operations	● Nurse Executive and Dean have limited or no relationship
	● Both are frequently kept apprised of activities by other members of the partnership	
	● Both travel to attend at least at one VANA national meeting held annually in Washington, DC	● Leader introduces administrative barriers to program progress (e.g., in carrying out alleged organizational policy constraints)
		● Has an adversarial relationship with program director(s)
*Create visibility of VANA program*	● VANA program has high visibility within institutions and community (e.g., logo on signs, lanyards, cups, pens, screen savers, informational spots developed for local television coverage)	● No attempts made to increase awareness of VANA, especially among nursing (i.e., VA staff, nursing school faculty and students
*Identify and address logistical barriers*	● Partnership leadership demonstrate flexibility in regard to interpretation of rules, regulations, and policies of institutions that would pose barriers	● Inadequate mechanisms to complete student paperwork prior to VA rotations
	● Maintain regular meetings in order to provide a forum to bring up challenges and barriers	● Rigidity in interpretation of rules and regulations, creating barriers (e.g., defining work hours)
		● Absence of open lines of communication between leadership of the two organizations
*Market VANA to appropriate audiences*	● Repeated efforts to develop awareness of VANA within the:	● No resources (e.g., available personnel, funds for flyers) for marketing program
	→Medical Center	
	→Local community	
	→University (including outside of the nursing school)	
*Facilitate intra-organizational operation*	● Presence of a program champion, a firm and ardent believer in the program, who is able to achieve the buy-in from within the leadership and faculty necessary for the program to develop	● Absence of program champion, in leadership positions in particular
	● Holds annual off-site retreats to facilitate team building	Lack of attempts to build cohesion (e.g., retreats, team-building exercises)
*Refill partnership positions as needed*	● Key partnership leaders are consistent throughout the Launch Year	● Frequent turnover in key leadership positions
	● If turnover of key leaders occurs, the positions are filled with persons very familiar with the project and its role responsibilities, and also who has the active support of other program participants	● Filling key leadership positions with persons unfamiliar with the program, or who are not supportive of some of its major objectives
		● Proposal authors are no longer at the institution by end of first year of operation
***Outputs***		
*Local recognition for VANA program*	● Formal events and meetings held that highlight VANA participation (e.g., recognition ceremonies, information seminars)	● Lack of awareness of the VANA partnership both within institutions and in the local community
	● Interest from other nursing schools to participate in a VANA-like program	● No effort to collaborate on VANA-related publications
*VA-CON co-authored publications*	● VA-based and nursing school faculty and leadership involved in development and submission of publications	● No effort to disseminate VANA-related products
*Perceived benefits by all stakeholders*	All key stakeholders perceive at least some benefit from VANA participation, such as:	Few stakeholders perceive any benefit from VANA participation, such as:
	***University:***	***University:***
	● Opportunity for expanded curriculum (new course/subject matter; addition of veteran and VA-specific content)	● Increased student enrollments and faculty positions not commensurate with level of perceived benefits
	● Decreased concern about finding clinical placement slots	***VA:***
	● Appreciation of clinical expertise of VA-based VANA faculty	● No value seen in increasing career opportunities for expert nurses
	***VA:***	● *Students:* Negative VA experiences negatively impact student impressions of VA
	● Increased unit staff and patient exposure to BSN-prepared students	***Veteran patients:***
	● Improved retention of current nursing staff, especially those with valuable experience and clinical expertise	● Occasionally feel overwhelmed by presence of large clinical groups of student nurses
	● Expansion of simulation lab use and capabilities	
	***Students*** **:**	
	● Increased awareness of veteran-specific needs	
	● Increased awareness of employment opportunities at the VA	
	***Veteran patients:***	
	● Appreciation of interactions with VANA students, especially those with military background	

### Goal 1: increasing faculty positions

As shown in Table 1, the data from the interviews and focus groups with the leaders, faculty, students, and nursing staff involved in the fifteen partnerships suggested that the following activities were critical to achieving first year progress toward the goal of increasing faculty positions:

- Defined scope of faculty roles
- Initiated training for the faculty role and for faculty development
- Planned to integrate faculty into the partnering environment
- Adjusted faculty workloads as needed
- Integrated faculty into the partnering environment
- Provided APRN faculty with protected time for patient care

For each activity, the indicators of success and challenge illustrate the variation in performance of the activities that ranged from “proceeding well according to plan” to “addressing impediments and therefore lagging behind schedule.”

We found that the scope of the faculty role varied widely amongst partnerships. Depending on the program structure, the distribution of full-time equivalents [FTEs] ranged from a full (1.0) FTE to less than half (e.g., 0.3 FTE) dedicated to the VANA faculty role. In situations where the dedicated FTE was below 0.5 FTE, commitment on the part of the faculty to the VANA program seemed diminished. Recognition of the need to provide faculty with “protected time” from teaching to engage in other activities such as faculty meetings, committee work, and patient care (for those needing to maintain their license as advanced practice registered nurses) suggested higher satisfaction rates on the part of the VANA faculty, particularly among those who were new to the faculty role.

With the emphasis on using expert clinicians who often had limited teaching experience as faculty, those partnerships who recognized early on the need to invest in developing these faculty had increased satisfaction of VANA faculty and decreased turnover during the first year. Furthermore, recognition of workload-life balance was a challenge for many partnerships. Oftentimes, the natural breaks in the school calendar (e.g., late December) that traditional faculty receive were not available to VA-based faculty because of their clinical responsibilities at the VA facility, thus not providing them with adequate respite between terms. Issues around traditional working hours for hospital-based staff versus the more flexible hours of traditional nursing school faculty often resulted in work overload issues for faculty.

Multiple challenges were noted around merging the organizational cultures of VA and university partners. Faculty from both sides viewed the other partner as a large, bureaucratic organization that created a number of difficulties around time-keeping for faculty. University-based faculty with academic experience uniformly described their academic culture as providing more flexibility than the VA in terms of where and when work is completed. Some VA employees expressed concern that this more lax approach to time-keeping with their university partner had the potential to limit accountability.

Table 1 also shows that the critical outputs of year one efforts towards increasing faculty were:

- Faculty hiring quota met by end of Launch Year
- Faculty satisfied with new roles

The successful expansion of nursing faculty and student enrollments was prescribed by the VANA grant (i.e. five faculty FTEs, either VA- or nursing school-based, per 20 additional students enrolled into the designated baccalaureate program over baseline), with a goal of hiring faculty first from the local VA facility, in order to encourage retention of clinically expert nursing staff within the VA system. Challenged programs often were unable to meet the hiring quota. When hiring quotas designated by the grant were not met by the end of the first academic year, it was generally due to a lack of qualified candidates in the local area. At some sites, the full quota was not filled during the initial year.

Satisfaction was measured primarily via faculty surveys, which looked at leadership support, mentorship, availability of teaching resources, and workload, among other issues. High dissatisfaction and turnover of faculty resulted primarily from unrealistic expectations in the first year of the partnership about the role and its associated workload.

### Goal 2: increasing student enrollment

As shown in Table 2, our qualitative analysis suggested that two implementation activities during the Launch Year were critical to progress towards this goal:

- Generated interest in VA clinical placements amongst students
- Placed students on VA facility units

The number of additional matriculating students was prescribed by the VANA grant in direct relation to the expansion of VANA faculty as indicated above. In rare cases, enrolling the target number of new students was not achieved, possibly due to a drop in applicants secondary to the poor state of the economy or the inability of the nursing school to accommodate that number of new students into coursework when new faculty were not yet recruited. Even if the increase in student enrollments was accomplished the first year, some partnerships encountered logistical problems related either to delays caused by complex hiring processes or difficulties transferring funds to the university to pay for nursing school-based positions.

Some VANA programs created cohorts of students who went to the VA facility for all of their clinical rotations (except pediatrics and obstetrics). Other programs chose to send most of their students to the VA for at least one clinical rotation. In both situations, efforts were made to take advantage of the unique opportunities of the VA, such as mental health and community health placements, in order to enrich the student experience. VA employment policies, like those of many practice institutions, were often cumbersome and could significantly delay placement of students onto units. Having processes in place prior to the start of the semester as well as adequate resources to process and orient the students allowed students to start their rotations within an appropriate time frame.

Two outputs appeared critical to this goal:

- Increased undergraduate student enrollment
- Increased student satisfaction with participation

Prior to VANA, students’ opinion of the quality of VA clinical rotations was often poor. After experiencing VA through VANA, students consistently reported high rates of satisfaction in surveys regarding their VA clinical rotations. Survey data will be reported elsewhere. A primary disappointment was the lack of job opportunities available at the participating VA facilities.

### Goal 3: implementing curricular innovations

Table 3 shows the implementation activities that emerged from interviews and focus groups as critical to first year progress towards the goal of curricular innovations:

- Operationalized proposed innovations
- Initiated proposed innovations
- Collaborated on research and quality improvement initiatives
- Refined program components as needed

Examples of innovative programs that were launched during the first year of operation included enhanced use of simulation learning, “embedding” VANA faculty members on certain units as expert resources even when they were not accompanied by students [18], designated educational units (DEUs), and evidence-based projects (EBPs) conducted collaboratively with students and unit staff. [5] Since most innovations often focused on aspects of the faculty role, providing resources for faculty to conduct their business was crucial. Office space, space for conducting regular VANA meetings, email accounts, textbooks for courses taught, and parking were resources that successful programs had in place at or near the start of the program. Implementing innovations as detailed in the partnerships’ proposals often lagged behind the proposed schedule at challenged sites. Successful innovations were implemented as planned despite challenges along the way.

Successful partnerships typically foresaw opportunities for combined scholarly activities that optimized available expertise and were built into the strategic plan from the beginning. EBP projects often became the focus of such collaboration, likely because these quality improvement projects could be better integrated into the educational curriculum and therefore were easier to initiate. Potential opportunities for shared research often centered on the expertise of a single person who had a track record of such work; as the development of research studies typically requires more than one year, it is not surprising that initial efforts aimed at fostering collaborative research focused on persons with significant pre-existing experience in research. Plans of this nature were frequently delayed by unanticipated Launch Year logistics, where challenged sites either delayed the implementation of such plans or did not develop them during the initial year.

Three outputs emerged as critical to this goal:

- Increased stakeholder satisfaction with participation
- Increased evidence-based care
- Perceived improvements in nursing care quality

Early on, new nursing students were not always welcomed by staff or patients, either because some VA facilities had not recently, or only infrequently, hosted baccalaureate students and accordingly did not know what to expect from them, or because some older staff nurses were reluctant to take on the role of preceptor. However, at successful sites unit nurses and, especially patients, were delighted and often enthusiastic about the clinical rotations and were impressed by the high caliber of the VANA students.

In successful partnerships, VANA afforded the VA partner with the opportunity to gain the benefit of faculty knowledge and experience to develop programs designed to foster the spread of evidence-based nursing care (e.g., development of a “wish-list” of programs where faculty choose to provide their expertise, bringing in nationally prominent visiting scholars to lecture at the VA partner). The partnerships’ efforts at joint research activities were limited in the Launch Year, likely because they were trumped by the logistics of program implementation. Where they existed, research collaborations benefited from the existence of VANA (e.g., development of a joint VA-university research position) resources.

### Goal 4: increasing recruitment and retention

Two activities appeared to be important to progressing towards the goal of increasing recruitment and retentsion:

- Identified VA units to be used for VANA placements
- Involved nursing staff in planning

Each of these activities were implemented with varying degress of success, as illustrated by the indicators of success and challenge presented in Table 5.

On some units, VA nurses served as role models or mentors for students as they sought to learn more about the nature of nursing practice and in what area they wished to practice. One partnership underscored this role by assigning formal mentors for students. Mentoring was reported as valuable both to the students and the nursing staff who served as mentors. Some respondents noted that by significantly increasing the length of time that the students were at a single clinical institution, students were more likely to view themselves as part of a team and were able to take on advanced responsibilities because they had a more detailed understanding of that institution. A few respondents noted that the opportunity for students to observe VA nurses in a variety of roles, including managerial positions, provided students with broader ideas for how their nursing careers could develop within VA. Challenged sites were characterized by staff resistance to having students, unwillingness by staff to engage in EBP-related activities, or over-reliance on staff for bedside teaching because of large clinical groups.

Outputs of the efforts to launch recruitment and retention included the following:

- Increased stakeholder satisfaction with participation
- Benefits of VANA participation realized

Unit nurses were the primary benefactors of VANA student and faculty presence because of a variety of factors. Oftentimes, we were told that staff prepared for students’ questions by refreshing their own knowledge and occasionally sought the expertise of the VANA faculty to do so. Clinical innovations implemented at some sites (e.g., DEUs, embedding faculty as unit staff) activated staff in ways not seen prior to the partnership launch. We frequently heard anecdotes about observed improvements in care quality. Sites where staff continued to perceive student placements as burdensome were considered to be challenged. Realized benefits of VANA participation included the provision of career opportunities for expert VANA nurses who wished to teach, well-qualified graduates seeking work at the VAMC, and a much improved reputation for the VA as a high quality and competitive clinical rotation.

### Goal 5: promoting collaboration

An underlying VANA goal was to promote collaboration between the academic and practice partners in order to build a strong, mutually beneficial relationship. A number of activities were important to achieve this goal (Table 5):

- Initiated communication structure
- Created partnership governance
- Elicited support for program from all levels of organizational leadership
- Delineated level of each Program Director’s involvement
- Delineated level of Dean’s and Nurse Executive’s involvement
- Created visibility of VANA program
- Identified and address logistical barriers
- Marketed VANA to appropriate audiences
- Facilitated intra-organizational operation
- Refilled partnership positions as needed

Formal and regular meetings that included the VANA faculty allowed for the development of a strong cohesive group of faculty who could rely on each other for support and encouragement. When these meetings included leadership from both organizations, and where leadership encouraged participation in committee meetings from both institutions, faculty perceived that their expertise was more highly valued by the partnering organization. Barriers arose when one or the other program director did not regularly attend the meetings, or if faculty felt that they were beholden to incongruent demands from both organizations. Shared decision-making and trust marked those partnerships considered to be successful.

Other innovations, while strongly encouraged, were not requirements of the grant. These new activities could focus on clinical care, administrative practices, research, or education. Programs that received VANA funding generally proposed realistic and robust innovations, although challenged programs often had to delay the implementation of these activities due to logistical barriers created by partnerships that did not have pre-existing clinical placement agreements with their VA partner and thus were not fully aware of the demands associated with such arrangements. Similar challenges were also created by difficulties in the timely backfilling of clinical and managerial responsibilities of those VA nurses who took faculty positions. Even successful programs occasionally experienced delays in launching proposed innovations because of the short length of time between the notification of grant funding and the beginning of the academic year. Examples of innovative programs that were launched during the first year of operation included enhanced simulation learning, embedding VANA faculty members on certain units as expert resource staff even when they were not accompanied by students, DEUs, and EBP projects conducted collaboratively with students and unit staff.

Overt attempts made to market the VANA partnership within each organization and within the community were viewed as a positive indicator of a partnership that was proud of its work. Pride and strong program identity were weak or absent at challenged sites.

Culture clashes and administrative difficulties were frequently encountered across all programs. Such hurdles, often unforeseen, were either dealt with efficiently without interrupting launch implementation, or were met with poorly coordinated problem-solving or a lack of necessary involvement from organizational leadership.

The consistency of core people in the partnership, particularly in the leadership “quadrad” (i.e. Dean, VA Nurse Executive, both Program Directors), was markedly emblematic of successful programs. In particular, having key personnel who took part in conceptualizing the program and writing the proposal remain associated with the program throughout the launch phase had greater value for maintaining morale and overcoming administrative and other challenges. Early turnover in faculty was viewed as highly disruptive to these nascent programs. Where small fragments of FTEs were assigned to VANA faculty roles, turnover was higher.

The following outputs emerged as critical to this goal:

- Local recognition for VANA program
- VA and academic partner co-authored publications
- Perceived benefits by all stakeholders

Events held within VAs and the larger nursing community were common. Knowledge of the VANA program was poor outside of the direct participants in the partnership at challenged sites.

Drafting manuscripts describing unique aspects of certain VANA-supported activities started early at successful sites, whereas sites that were still struggling to overcome logistical problems in their first year, such as delays in faculty hiring, were challenged to meet the terms of the grant and unable to develop scholarly products related to the partnership.

Benefits of VANA program involvement were recognized at all levels of stakeholders at successful sites. In particular, the value of adding content regarding veteran care to the nursing curriculum (most commonly as vignettes in the simulation learning settings) and the positive feedback from veteran patients who received care from the students were notable. Discussions about the value of VANA were infrequent at sites, especially at sites where VA-based VANA faculty were poorly integrated into the nursing school environment and where unit staff at the VAMC were resistant to having students on their unit.

### Initial program inputs and environmental context

The types of resources invested in the individual programs were similar across all partnerships. Some of the model’s inputs were anticipated in the original grant, including the funds to hire new faculty, but others were added to the framework as they emerged from the data. As shown in Table 6, important inputs included:

**Table 6:** **Critical elements: initial program inputs**

**Critical elements**	**Indicators of success**	**Indicators of challenge**
***INPUTS***		
*Participation in proposal development*	● Dean, Nurse Executive, and both program directors participated in proposal writing	● One or more key leadership positions were vacant at time of proposal preparation
	● Some future faculty candidates produced specific content regarding proposed innovations	● No input from clinical faculty or nursing staff included in proposal content
*Collegiality between future partnership members*	● Longstanding pre-existing relationships between two or more partnership leaders	● One or more key leaders met for first time during proposal development or after grant notification
*Relationship of partnership leaders with VA Central Office*	● Longstanding pre-existing relationships between partnership leaders and VA’s Office of Academic Affiliations	● Leaders of proposed partnerships were unknown to OAA staff prior to submission
*Funds from VANA grant to support faculty salaries*	● Clear understanding at both institutions about procedures to disburse VA grant funds for faculty salaries	● Pervasive lack of knowledge at both institutions about the local financial mechanisms used to spend grant funds
		● Lack of knowledge about grant disbursement process or lack of supportive resources to understand it

- Leadership participation in proposal development
- Collegiality between future partnership members (often based on prior work together)
- Relationship of partnership leaders with the VA Central Office
- Funds from VANA grant to support faculty salaries^a^

Participation in proposal development was an important indicator of a stakeholder’s level of buy-in to the partnership’s mission, and seemed to be predictive of a sustained commitment to VANA throughout the Launch Year. It may also have assured full consideration of operational issues that had to be addressed in the ramp-up of the program, and led to a speedier and smoother implementation process.

Pre-existing relationships between leaders showed a commitment to the program between professionals who had worked together in the past. When such a relationship existed, it suggested a higher level of communication between partners, which not only facilitated informal, *ad hoc* contacts during the critical developmental phase of the nascent partnership, but also formed the basis for formal communication structures that facilitated the program’s overall success.

If partnership leaders were known to VANA directors in the VA Office of Academic Affiliations prior to receiving the VANA grant, that familiarity seemed to facilitate informal and therefore timely communication with that Program Office, and appeared to be especially beneficial when the partners confronted logistical barriers. Challenging problems were dealt with earlier when a Program Director or Dean, for example, felt comfortable contacting a colleague from the “third-party” VA Program Office to seek advice or assistance. Leaders in partnerships that did not have that relationship either looked to the partnership network for advice or attempted to cope with problems amongst themselves.

The primary input of the VANA program was funding provided specifically for the expansion of nursing faculty and baccalaureate students, conducted on a graduated basis. New faculty FTE were appointed based on a fixed ratio of new faculty FTE to additional nursing students enrolled over baseline prior to the start of VANA at the partnering nursing school. The prescribed ratios increased according to enrollment guidelines established in the pilot program, and decreased again in the final year of the program as the pilot neared its end.

Additionally, a number of important environmental features appeared to meaningfully impact a partnership’s operation. For example, the economic downturn factored significantly in all cases, but with widely varying impacts on local programs. In some instances where the academic partner was a state-supported institution, the VANA grant provided key funding for struggling nursing schools. In general, balancing the needs of both partners was well managed in most cases. The most common contextual features that we found were:

- Local competition for undergraduate clinical placement sites between nursing schools
- Local availability of qualified faculty candidates
- Effect of the local economy on employment

If several schools of nursing, including baccalaureate and community college programs, existed within a particular locale, we frequently observed competition for clinical placement slots. Commitment to partnering with VA through the VANA grant sometimes pre-empted such agreements where they had previously existed. Despite the demand for clinical places, we also observed that many VA facilities became clinical placements sites for their nursing school partners for the first time.

The search for clinically expert, masters-prepared VA nurses who wanted to develop themselves as educators was easiest where there was a plentiful supply of nurses with master’s degrees in the surrounding community. We most commonly observed that VANA faculty positions were offered to qualified VA nurses. However, in locales with a short supply of those nurses, VA facility leadership was often reluctant to release these valuable employees out of concern that the facility would be unable to backfill their positions. Filling the first year hiring quota was often delayed as partnerships were forced to search outside the immediate area for qualified applicants. In turn, this often delayed the program’s launch.

We found that local economic conditions impacted the VANA program in several ways. The most apparent effect was the (temporary) absence of a nursing shortage and the resulting lack of demand for new nurses. In general, the economic downturn since 2007 has altered the dynamics of the nursing shortage and, as such, there were fewer opportunities for VANA graduates to seek VA employment, especially in urban areas where the competition for jobs was most intense. As the economy recovers, it is anticipated that demand for new nurses will increase [19], but until that happens, fulfilling this aim of the VANA pilot program was not considered feasible. Hiring freezes and delayed retirements across VA facilities were common. In at least a third of the partnerships, VANA graduates who entered a nursing program with the idea that they would find work opportunities at a VA facility, were frequently disappointed to find few or no job openings.

## Discussion

In this systematic evaluation of a nationwide academic-practice partnership program conducted during the VANA program’s Launch Year, we identified indicators of both successful and challenged launches. These indicators are the results of our observations obtained during the launch of the 15 ongoing academic-practice partnerships that were established between nursing schools and proximally-located VA medical centers. VANA’s nationwide breadth combined with the variation in the structure of each individual VANA partnership, allows this study to provide more generalizable information on academic-practice partnerships than prior studies that have been limited to small samples in single locations [6].

Five key themes emerge from this analysis. First, inter-organizational collaboration (“teamwork”) is a critical factor in enabling these partnerships to be successful. Such collaboration would include teamwork between direct and indirect participants across partnering institutions regarding specific issues, strategies, and decisions. Factors that facilitate building strong collaboration include prior working relationships and personal contact between leaders of the partnering organizations (often borne from preexisting clinical education relationships), a sense of equal participation and equitable burden on partners, clear and realistic expectations of the benefits and responsibilities of each party, and building ongoing and frequent opportunities for formal and informal communication between program participants at all levels. These processes were challenged when leaders failed to commit the time and effort to establish and maintain the collaboration, when informal communications were weak, when formal meetings and communications were sporadic or limited, and when commitments such as achieving enrollment targets or providing clinical instructors or preceptors were not fully met. In the VANA program, developing a proposal for funding from VA provided a vehicle for defining how the collaboration would be structured and time to consider issues before implementation began. In other contexts, where no such external mechanisms exist, the experience of the VANA sites suggests that formal, intensive planning processes will be invaluable to successful implementation.

Second, major challenges to creating the partnerships arose not just from blending different cultures, but also from having to integrate activities across divergent organizational processes and constraints. In the VANA context, for example, time keeping requirements and expectations about working on site for those working directly for the VA facility (as opposed to those working at schools of nursing) clashed with the nursing school’s expectations about time commitments across and between semesters. Similarly, nursing faculty expected greater flexibility to prepare for classes and grade assignments off-campus. Not all these challenges were anticipated during the planning stages, and sites differed in the speed with which these challenges were identified and addressed. In the VANA program, subsequent cohorts learned from earlier cohorts and better anticipated these challenges. The issues of operational integration identified in Table 1 should be expanded and extended to examine the experience of non-VA partnerships to see whether they differ from what we observed.

Third, there are specific challenges associated with identifying and recruiting nurses in clinical settings to take on faculty roles in a timely fashion, expanding the number of students recruited to programs when additional faculty are made available, and scheduling clinical and didactic courses. In the VANA program, these challenges were common across a number of sites. In some cases, these challenges proved daunting and substantially weakened the partners’ commitment to the success of the partnership. Expectations about ramp-up time need to be realistic, and operational problems should be closely and continually reviewed on a joint basis by partnership leadership.

Fourth, expectations for these partnerships extended beyond merely building faculty or increasing the number of students and included such activities as using nursing school faculty to improve the use of EBP in clinical settings or increasing the use of simulation-based learning for in-service training of nursing staff. Oftentimes, planning for these activities and how they would be implemented was much less well developed than were the plans for expanding clinical and didactic education. Implementation was thus more inconsistent. Partnership planning and management need to be more explicit and detailed about how these components of a partnership are to be implemented. Nevertheless, based on the partnerships that we observed, it would appear that such efforts are better aimed at implementation after the Launch Year in a sequential planning and development process. Our findings contain lessons that can be applied to these efforts. Moreover, this theme suggests that academic-practice partnerships may be an effective means to achieve the full involvement of nurses in efforts to improve practice environments and outcomes of care as called for by the Rob Wood Johnson Foundation Initiative on the Future of Nursing in its 2011 report [20].

Finally, the immediate drivers for the VANA initiative – a critical nursing shortage, the desire of the VA to address it by adding clinical nursing faculty, and VA’s goal of increasing nursing student interest in employment at VA facilities – have been tempered by the economic downturn and the temporary easing of the nursing shortage. Nonetheless, partnerships are long term commitments for both clinical sites and nursing schools. Partners need to find ways to build stable relationships based on long-term interests and commitments even as they adjust to short-term changes in the supply and demand for nursing care.

The present study focuses on the critical elements of a successful academic-practice partnership launch, as informed by three successive annual cohorts. As the partnerships continued to evolve after the first year, it became evident that, as expected, partnerships responded in various ways to the identified challenges. Therefore, while we believe that the launch year is critical to a program’s eventual success, it should be recognized that partnerships evolve over time by confronting existing challenges and responding to new challenges. For VANA, this organic process benefited from interactions between the partnerships and also from the involvement of VA Headquarters personnel. Future studies will examine the growth of the partnerships and address the long-term effectiveness, efficiency, and sustainability of the VANA program.

The AACN-AONE Task Force on Academic-Practice Partnerships recommended a number of principles to guide such partnerships. These principles include collaborative relationships, mutual respect and trust, and shared knowledge, as well commitments to maximizing the potential of all nurses, effectively transitioning them into practice, highlighting their academic achievements, redesigning practice environments, and analyzing the needs of the nursing workforce [15]. While aligned with these principles, our findings suggest more specific implementation strategies for launching academic-practice partnerships. The critical implementation activities and outputs that emerged from our research provide a more basic level of operational guidance for implementing such partnerships, although some, such as those critical for promoting collaboration (Table 5), directly address a Task Force principle (collaborative relationships) as well.

While the framework we present can serve as a guide to best practices for establishing academic-practice partnerships [6,21,22], some limitations to our study should be acknowledged. First, each of the domains that influence partnership performance includes observations from our site visits that we believe delineate whether a partnership’s performance can be characterized as successful or challenged. The indicators of relative success of a partnership in a particular domain are based on our actual observations and not necessarily indicative of ultimate success in the latter stages of partnership development. Second, each of the domains represents different influences on performance and should be considered independently. In other words, partnerships could be viewed as successful or challenged in one or more of these domains, but when all of the domains are considered together, the overall partnership could still be considered to have largely met VANA’s programmatic goals overall. Finally, all of the studied partnerships include only VA as the practice partner. Thus, our results may not generalize to other practice partners due to dissimilarities of VA healthcare institutions compared to others in the same communities.

Although our evaluation examined academic-practice partnerships within nursing, it is likely that our framework would be applicable to academic-practice partnerships that involve other clinical practitioners. Future work should examine whether this Critical Elements approach is applicable to partnerships that include other types of healthcare professionals.

## Conclusion

Developing an academic-clinical partnership requires identifying how organizations with different leadership and management structures, different responsibilities, goals and priorities, different cultures, and different financial models and accountability systems can bridge these differences to develop joint programs integrating activities across the organizations. This can be a daunting task. Furthermore, understanding how these partnerships emerge, and are nurtured and challenged also requires adopting a conceptual model in which partnerships can evolve over time as they learn from experience and attempt to address operational challenges. The experience of the VANA sites in implementing academic-clinical partnerships provides a broad set of experiences from which to learn about how such partnerships can be effectively implemented, the barriers and challenges that will be encountered, and strategies and factors to overcome challenges and build an effective, sustainable partnership. The findings provide practicable, actionable guidance on strategies for successfully launching academic-practice partnerships in nursing. By paying attention to the critical implementation activities and outputs identified here, decision-makers will be able to make better informed decisions about how to structure an academic-practice partnership for greatest success during the critically important initial year of the partnership’s creation.

## Endnote

^a^Full details of the grant requirements of the VANA partnership model are described in Bowman et al., [5].

## References

[1] Isoraite M (2009). Importance of strategic alliances in company’s activity. Intellect Econ.

[2] Ellison G (2008). “Blowing open the bottleneck” in Oklahoma. Okla Nurse.

[3] Brown D, White J, Leibbrandt L (2006). Collaborative partnerships for nursing faculties and health service providers: what can nursing learn from business literature?. J Nurs Manag.

[4] Cronenwett LR (2004). A present-day academic perspective on the Carolina nursing experience: building on the past, shaping the future. J Prof Nurs.

[5] Bowman CC, Johnson L, Cox M, Rick C, Dougherty M, Alt-White AC, Wyte T, Needleman J, Dobalian A (2011). The Department of Veterans Affairs Nursing Academy (VANA): forging strategic alliances with schools of nursing to address nursing’s workforce needs. Nurs Outlook.

[6] Beal JA (2012). Academic-service partnerships in nursing: an integrative review. Nurs Res Pract.

[7] Delunas LR, Rooda LA (2009). A new model for the clinical instruction of undergraduate nursing students. Nurs Educ Perspect.

[8] Erickson JM, Raines DM (2011). Expanding an academic-practice partnership. J Prof Nurs.

[9] Horns PN, Czaplijski TJ, Engelke MK, Marshburn D, McAuliffe M, Baker S (2007). Leading through collaboration: a regional academic/service partnership that works. Nurs Outlook.

[10] Kowalski K, Horner M, Carroll K, Center D, Foss K, Jarrett S, Kane LA (2007). Nursing clinical faculty revisited: the benefits of developing staff nurses as clinical scholars. J Contin Educ Nurs.

[11] Murray TA (2007). Expanding educational capacity through an innovative practice-education partnership. J Nurs Educ.

[12] Murray TA (2008). An academic service partnership to expand capacity: what did we learn?. J Contin Educ Nurs.

[13] Warren JI, Mills ME (2009). Motivating registered nurses to return for an advanced degree. J Contin Educ Nurs.

[14] Wellard SJ, Williams A, Bethune E (2000). Staffing of undergraduate clinical learning programs in Australia. Nurse Educ Today.

[15] **Academic Practice Partnerships.** [https://www.aacn.nche.edu/leading-initiatives/academic-practice-partnerships]

[16] Stetler CB, Legro MW, Wallace CM, Bowman C, Guihan M, Hagedorn H, Kimmel B, Sharp ND, Smith JL (2006). The role of formative evaluation in implementation research and the QUERI experience. J Gen Intern Med.

[17] Parker LE, Kirchner JE, Bonner LM, Fickel JJ, Ritchie MJ, Simons CE, Yano EM (2009). Creating a quality-improvement dialogue: utilizing knowledge from frontline staff, managers, and experts to foster health care quality improvement. Qual Health Res.

[18] Rittman M, Hinze M, Citty S, Chappell J, Anderson C (2010). The VA nursing academy embedded clinical education model. Nurse Educ.

[19] Buerhaus PI, Auerbach DI, Staiger DO (2009). The recent surge in nurse employment: causes and implications. Health Aff (Millwood).

[20] Committee on the Robert Wood Johnson Foundation Initiative on the Future of Nursing at the Institute of Medicine (2011). The Future of Nursing: Leading Change, Advancing Health.

[21] Beal JA, Breslin E, Austin T, Brower L, Bullard K, Light K, Millican S, Pelayo LW, Ray N (2011). Hallmarks of best practice in academic-service partnerships in nursing: lessons learned from San Antonio. J Prof Nurs.

[22] Teel CS, MacIntyre RC, Murray TA, Rock KZ (2011). Common themes in clinical education partnerships. J Nurs Educ.

